# Effects of Acute Tryptophan Depletion on Repetitive Behavior in Laying Hens

**DOI:** 10.3389/fvets.2019.00230

**Published:** 2019-07-11

**Authors:** Patrick Birkl, Jacqueline Chow, Peter McBride, Joergen B. Kjaer, Wolfgang Kunze, Paul Forsythe, Alexandra Harlander-Matauschek

**Affiliations:** ^1^Department of Animal Biosciences, University of Guelph, Guelph, ON, Canada; ^2^Friedrich-Loeffler-Institute, Federal Research Institute for Animal Health, Institute of Animal Welfare and Animal Husbandry, Celle, Germany; ^3^Department of Medicine, Brain-Body Institute and Firestone Institute for Respiratory Health, McMaster University, Hamilton, ON, Canada

**Keywords:** domestic birds, feather pecking, operant chamber, ATD, cognition

## Abstract

Repetitive pecking at the feather cover of other birds (FP) is one of the most important welfare problems in domestic birds. It is not only characterized by motor symptoms, but also by an innate vulnerability of the serotonergic system. Moreover, the serotonergic system influences cognitive function. Acute tryptophan depletion (ATD) is a widely used method for studying serotonergic function in mammals and has been recently validated in birds. However, a tryptophan-deficient amino acid mixture has never been tested on groups of birds to impact their social behavior, including repetitive feather pecking, nor has it been given to potentially impact their cognition and motor performance. One hundred and sixty White Leghorn laying hens consisting of two genetic lines divergently selected to perform high (H) or low (L) levels of FP, and an unselected control line (UC), were kept in 10 groups consisting of 4 H, 3 L, and 9 UC genotypes. In a counterbalanced order, half of the groups were first subjected to an ATD treatment, while the other half were first given a balanced control (BC) treatment, and vice versa, after which their feather pecking behavior was observed. The effect of ATD/BC on repetitive pecking, motor performance, and cognition was investigated in a 5-s delayed reward task in an operant chamber with 10 phenotypic feather peckers, 10 recipients of feather pecking, and 10 bystanders (who neither performed nor received feather pecks). ATD given to groups of birds induced gentle, repetitive feather pecking in all genotypes. Following ATD, phenotypic feather peckers performed more poorly during the delayed reward task, as seen by their higher number of repetitive, non-rewarded key, and non-key pecks in the operant chamber. In conclusion, ATD impacted the hens' social behavior by increasing the number of repetitive gentle feather pecks at conspecifics. Furthermore, feather peckers were more likely to peck while waiting for a reward after ATD, suggesting a role for the serotonergic system on cognition in these birds.

## Introduction

Birds kept for egg-laying engage in a variety of social interactions. However, some behaviors can become problematic, such as excessive aggressive pecking and feather pecking (1). Feather pecking (FP) is characterized as an interaction between two birds, with one bird pecking repetitively at a conspecific's feather cover (2). This behavior may include the removal and ingestion of feathers, resulting in loss of feather cover, skin damage, or death (3).

Ethologists consider FP to be an expression of frustration arising from a lack of adequate opportunity to perform foraging behavior (4). However, FP is also known to occur even when birds are provided with outdoor access to accommodate foraging (5). Furthermore, FP birds seem to lack behavioral control, pecking functionlessly, and repetitively during operant tasks (6). Considering these factors and findings, FP is more akin to abnormal repetitive behavior, similar to those seen in human psychiatric disorders, such as autism-spectrum disorders (ASD), attention-deficit-hyperactivity disorder (ADHD), and schizophrenia (7, 8).

There is some evidence that humans with the above-mentioned psychiatric disorders also show abnormalities in the serotonin (5-HT) system (9). Additionally, the biological basis of repetitive behavior may include the modulatory role of 5-HT, which is involved in various forms of motor activity and co-occurring cognitive problems (10–12). Interestingly, the performance of repetitive motor behavior increases 5-HT neuronal activity in mammals, which may lead patients with the above-mentioned disorders to engage in such behavior as a means of self-medication (13). Similarly, it has been suggested that the avian 5-HT system (14) and the precursor tryptophan (TRP) are intimately linked to repetitive FP. Furthermore, genetic factors (15) such as candidate genes linked to the 5-HT system (16), lower forebrain 5-HT turnover levels (8, 17), and nutritional factors, including low dietary TRP (18) also point toward a serotonergic involvement in the development of FP behavior.

Since 5-HT cannot cross the blood-brain barrier, central 5-HT synthesis depends on the availability and transport of its precursor, TRP, in the brain (19). TRP competes with other large neutral amino acids (LNAAs) for active transport by the large amino acid transporter system (20). Consequently, decreased peripheral TRP relative to other LNAAs results in a lower TRP influx into the central nervous system. This phenomenon is demonstrated by the dietary acute tryptophan depletion (ATD) method, in which an orally-administered amino acid mixture lacking TRP that reduces 5-HT levels in the brain (21, 22) is frequently used as a non-invasive research tool to assess the involvement of the 5-HT system in human psychiatric disorders. To this end, Birkl et al. (23) showed for the first time that an ATD mixture in laying hens effectively reduces the ratio of TRP to all LNAA by 70% of baseline levels, 3 h after administration.

Although it was previously shown that the 5-HT system might modulate the social behavior problem of FP in laying hens (24), to our knowledge, no one has performed an ATD challenge on a group of birds by giving them a tryptophan-deficient amino acid mixture. Based on the hypothesis that ATD might increase antagonistic behavior between human individuals and those they interact with young (25), we designed our first experiment and hypothesized that ATD would increase repetitive FP in laying hens under group housing conditions.

The second experiment, which involved a modified, delayed reward task in an operant chamber, was aimed at comparing repetitive pecking at a pecking key by individuals categorized based on their phenotypic FP behavior before and after ATD treatment. Human studies have shown that participants perform more poorly with a dysphoric distractor in a proofreading task after ATD treatment, possibly due to ATD causing a shift in attention to the distractor (10). As such, we hypothesized that ATD-treated birds would shift their attention to a dysphoric or frustrating stimulus (a 5-s delay in reward dispension), as shown by increasing the number of repetitive pecks at the pecking key during the delay period. Finally, we hypothesized that this effect of ATD would be most pronounced in hens phenotypically identified as active feather peckers.

## Materials and Methods

### Ethical Statement

This study was approved by the University of Guelph Animal Care Committee before testing (Animal Utilization Protocol # 3206).

### Animals, Housing, and Treatments

Birds with a tendency to engage in FP were required for this experiment. One hundred and sixty non-beak-trimmed White Leghorn laying hens were raised in 10 identical aviary pens (16 birds per pen) at Arkell Research Station at the University of Guelph. The high (H) and low (L) FP and unselected control birds (UC) in the present study originated from a selection experiment (15) in which birds of one line were divergently selected based on high and low levels of FP behavior. Each pen contained 4 H, 3 L, and 9 UC line birds. Birds were kept in identical pens littered with wood shavings (9 birds/m^2^; 15 cm perch length/bird at 90 cm above the ground; 125 × 31 cm platforms 65 cm above the ground; 120 cm^2^ nest/bird; 10 cm food trough length/bird; 10 nipple drinkers). Cameras (Samsung SNO-5080R, IR, Samsung Techwin CO., Gyeongi-do Korea) were mounted on the ceiling above the entrance of each pen. Food (commercial laying hen mash) and water were provided *ad libitum*. The hens were kept in a ventilated, windowless room, on a 14:10 h light:dark cycle, with a light intensity of 25 lx at animal level, and an average daily room temperature of 20°C. The pens were separated by opaque boards to prevent physical and visual contact with birds from other pens [see Kozak et al. (26), for more details on housing].

### Operant Chamber Test Equipment

The custom-made operant chamber and computer (Med Associates, St. Albans, VT, USA) used in this experiment were kept in a testing room a short distance from the home pens. The polycarbonate operant chamber measured 52 L × 50.5 W × 56 H cm. One lighted pecking key (red light) was presented 36 cm above the ground (2.5 cm diameter). The feeder was accessible through a rectangular hole (13.5 L × 4.5 H cm) in the center of the test panel wall, 25 cm above the floor. The feeder was covered by a lid, which was manually opened and closed by the experimenter; food was only accessible when the lid was open. A LED house light was placed on top of the box to indicate the start of a trial and would automatically turn off at the end of each session. The operant chamber was controlled using the Trans IV program (Med Associates, St. Albans, VT, USA). The number of rewards received and the number of pecks on the pecking key were automatically recorded and saved. A camera (JVC GC-PX100BU HD Everio) was set up in front of the operant chamber to record each session.

### Acute Tryptophan Depletion and Balanced Control Capsules

The amino acid mixtures used for ATD and Balanced Control (BC) treatments were composed of the following 12 amino acids (Evonik, Essen, Germany): Arginine (0.145 g), Cysteine (0.081 g), Glycine (0.208 g), Histidine (0.035 g), Isoleucine (0.135 g), Leucine (0.17 g), Lysine (0.145 g), Methionine (0.062 g), Phenylalanine (0.098 g), Threonine (0.098 g), Tyrosine (0.08 g), Tryptophan (0.033 g for Balanced Control, 0 g for ATD), and Valine (0.145 g). The resulting mixture of 1.435 g was homogenized and aliquoted into two 1 g gelatin capsules (1.5 × 0.5 cm). For more details, see Birkl et al. (23).

### Tryptophan–Deficient Amino Acid Mixtures to Groups of Laying Hens—Feather Pecking

Five weeks before the experiment, the birds (24 weeks of age) were fitted with silicone “backpacks,” which consisted of two silicone squares (14.5 × 6 × 0.2 cm) resting on the back of the bird, secured with two plastic-coated clothesline wires which looped under the wings and attached to the silicone via eyelets (27). Each hen backpack was assigned one number for identification purposes.

**Figure 1:**
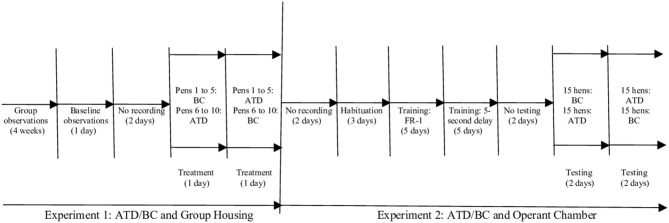
Schematic representation of experimental timeline for the acute tryptophan depletion (ATD)/balanced control (BC) group housing and operant chamber testing schedule.

We used gelatin capsules (CapsulCN® Int. CO., LTD size 00; cubage 0,95 cc), filled with a mixture of hard-boiled, chopped eggs, and commercial feed to habituate the birds to swallowing the capsules. As outlined in Figure 1, laying hens were treated (ATD/BC) and observed (baseline/treatment) over a period of 1 week. Pens were assigned to a counterbalanced order of capsule consumption: pens 1–5 received two ATD capsules first, and pens 6–10 received two BC capsules first. The ATD/BC capsules were administered to all hens, beginning at 7:00 h. The time of capsule administration for each pen was recorded to ensure that video analysis would commence exactly 3 h after capsule administration.

All occurrences (28) of FP interactions from the videos were observed and classified as gentle (repeated pecks at the tips and edges of feathers without removal) or severe (forceful pecking and/or removal of a feather) (2). Repeated pecks directed at the same individual were recorded as one bout. About ended when there were no pecks for 4 s (29). The instigator and receiver of each interaction were noted. Baseline (10 min) and ATD/BC (10 min) video observations took place 3 h post-capsule administration, which is when peripheral TRP depletion peaks (23). Video observers were blinded to the treatments.

### Tryptophan–Deficient Amino Acid Mixtures to Laying Hens in an Operant Chamber—Operant Pecking

Hens were selected for operant chamber testing based on their FP phenotypes. For phenotyping, birds were observed in their home pens twice a day (10 min between 9 a.m. and 11 a.m., 10 min between 2 p.m. and 4 p.m.), two times per week, over a period of 4 weeks. A feather pecker (P) was defined as a hen that delivered gentle and/or severe feather pecks more than 10 times in at least two 10-min video sessions and received fewer pecks than the number of pecks they delivered. A recipient was defined as a hen that received more than 10 pecks in at least two 10-min video sessions and pecked less than three times in any 10-min video session. A bystander was defined as a hen that neither pecked nor received pecks in two subsequent 10-min video sessions. Consequently, we created three distinct groups based solely on phenotype: 10 feather pecker birds (5 UC, 3 L, 2 H), 10 bystander birds (8 UC, 2 H), and 10 recipients (7 UC, 2 L, 1 H).

After habituating the 30 chosen birds to the operant chamber for 3 days, birds were then trained on a fixed ratio (FR) 1 schedule of reinforcement for another 3 days, whereby they pecked at an illuminated red key for immediate delivery of a high-quality food reward (chopped, hard-boiled egg). Birds were not food-deprived for habituation, training, or testing. One training session consisted of 20 rewards. To pass the FR 1 training phase, birds needed to meet the criterion of being able to peck the key and subsequently receive the food reward 15 times (75%) in a row on two consecutive sessions. All birds surpassed the training criteria. After the FR 1 immediate-reward sessions were completed, all hens participated in 5-s delay sessions (30).

**Figure 2:**
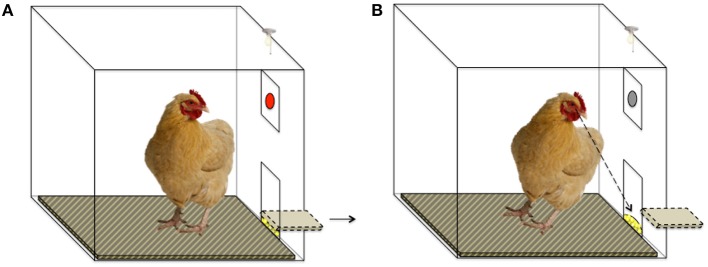
Schematic of Operant Chamber set-up. **(A)** The red key is illuminated, and a key peck will result in food reward after a 5-s delay. The food trough is in a closed position. **(B)** The food trough is opened after a 5-s delay and the hen can access the food reward. The red key is not illuminated. Key pecks at the un-illuminated key do not result in food reward.

**Figure 3:**
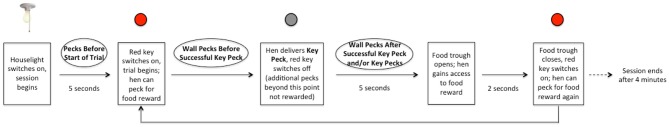
Timeline of 5-s delay schedule of reward.

Each session lasted 4 min (in which the maximum number of theoretically obtainable rewards per session was 32), during which a key peck resulted in a 2-s-long period of reward delivery, only if 5 s had elapsed from the key peck without reward (Figures 2, 3). All birds learned this task before the ATD/BC testing phase to control for learning effects.

Starting at 7 am during the ATD/BC treatment days, half of the birds (15) were given two ATD capsules, and the other half were given two BC capsules, with a 15-min delay between each bird (23). Testing on the 5-s delay schedule was performed at the point of maximal tryptophan depletion (3 h post-capsule administration).

The number of times the pecking key became illuminated and the number of pecks delivered at the key by each hen for both the FR 1 immediate-reward and 5-s delay schedules were recorded automatically by the MED-PC IV Software. Additionally, all hens were videotaped during all sessions to count the number of non-key pecks (i.e., pecks directed at the wall surrounding the key).

### Statistical Analysis

To analyze pecking behavior after dietary ATD vs. a BC treatment in the home pens, we used a Proc Glimmix procedure in SAS (SAS Institute Inc., Cary, NC 2016) based on the mixed modeling approach for randomized experiments. We analyzed the effect of treatment (ATD/BC) and line (H, L, UC), and their interactions on pecking behavior (gentle FP and severe FP bouts performed by each individual in 10 min) as the response variable, with baseline levels of FP behavior prior to treatment as a covariate. Data were fitted with a Poisson distribution. Due to repeated measurements being taken on the same group of animals and their pens, an autoregressive covariance structure of order 1 was fitted. The degrees of freedom were adjusted using the Kenward-Roger method.

To analyze the number of key pecks during the FR 1 and 5-s delay schedules, as well as the number of non-key pecks, we used a Proc Glimmix procedure with treatment (ATD, BC) and phenotype (pecker, recipient, bystander) and their interactions as fixed effects, while line, day of testing, and individual were used as random effects. Later, as no effect of day of testing was found, we removed this from the model. We used baseline pecking during the 5-s delay task performed by each individual as a co-variate. The data were fitted using a Poisson distribution. Estimated means ± standard errors of pecks per minute are reported.

## Results

### Effects of ATD/BC on Feather-Pecking Behavior in Group Housing

**Figure 4:**
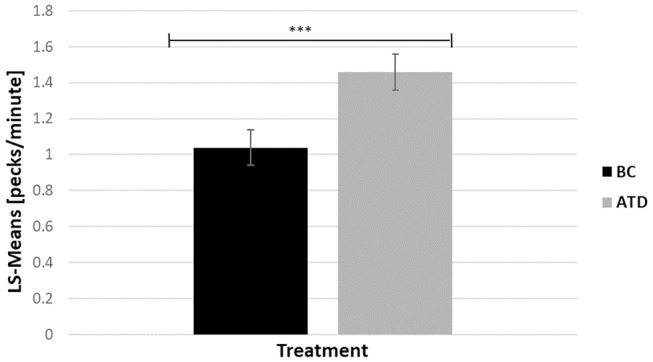
Least square-means (pecks/minute) of feather pecking behavior in acute tryptophan depletion (ATD)—treated birds and in Balanced Control (BC)—treated birds. Statistically significant results are indicated as ^***^P < 0.001.

There was a significant effect of dietary treatment, in which ATD-treated birds performed 28.77% more gentle FP bouts than BC birds [*F*_(1, 313)_ = 7.85, *P* < 0.005] (Figure 4). There was no effect of line and no interaction between dietary treatment and line on the number of gentle FP bouts. Severe FP hardly occurred during home pen observations. Therefore, no meaningful statistical analysis could be performed with respect to this behavior.

### Effects of ATD/BC on Performance in the Operant Chamber

Neither significant dietary treatment [*F*_(1, 1)_ = 1.18, *P* < 0.5; ATD: 3.07 ± 0.0 vs. BC: 2.98 ± 0.06 pecks/min] nor phenotype [*F*_(2, 1)_ = 0.23, *P* < 0.8; *P*: 3.06 ± 0.07; R: 2.99 ±0.07; B: 3.04 ± 0.07 pecks/min] effects, nor interactions [*F*_(21, 1)_ = 0.138, *P* < 0.9] were observed for the number of rewarded key pecks (= rewards obtained).

**Figure 5:**
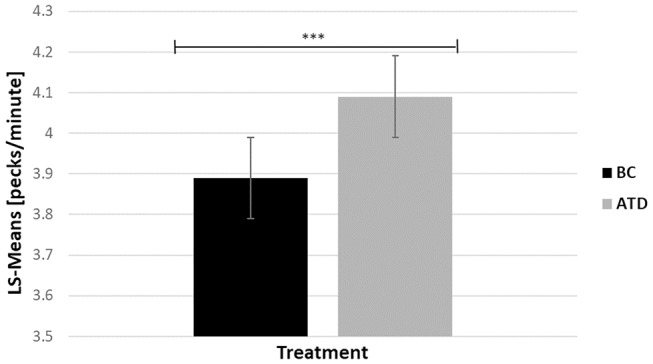
Least square-means (pecks/minute) of key pecks during the 5-s delay before a food reward was released in acute tryptophan depletion (ATD)—treated birds and in balanced control (BC)—treated birds. Statistically significant results are indicated as ^***^P < 0.001.

There was a significant dietary requirement effect in which ATD-treated birds performed 4.89% more 5-s delay key pecks than BC birds [*F*_(1, 213)_ = 12.87, *P* < 0.0017] (Figure 5).

The number of 5-s delay key pecks were significantly impacted by the treatment and phenotype interaction [*F*_(21, 21)_ = 24.51, *P* < 0.0001], where P hens delivered the highest number of 5-s delay key pecks during ATD, and the lowest number of pecks during BC treatment (4.37 ± 0.19 vs. 3.61 ± 0.21; *t* = −7.89, *P* < 0.0017), and vice versa in recipient birds (3.82 ± 0.21 vs. 4.13 ± 0.23; *t* = 2.40, *P* = 0.03). Bystanders were unaffected by the treatment (4.07 ± 0.21 vs. 3.92 ± 0.2; *t* = −1.41, *P* = 0.17).

## Discussion

To the best of our knowledge, the present study is the first to examine the effect of ATD treatment on (1) FP behavior of laying hens selected for high and low propensities of FP in a group setting, and (2) the key pecking performance of feather peckers (P), pecking recipients and bystanders in an operant chamber with a delayed reward schedule.

In the home pens, ATD treatment increased the overall number of gentle FP bouts for all genotypes of the hens. Severe FP was not triggered at all after ATD compared to the BC treatment. This result supports part of our hypothesis and confirms studies by van Hierden et al. (8) and Savory et al. (31) that the 5-HT system has a role to play in gentle FP behavior. Riedstra and Groothuis (32) stated that severe FP is embedded in bouts of gentle FP, and that there may be a common underlying motivation and/or neurobiological basis between these two forms of repetitive pecking. However, even though imbalances in the 5-HT system may constitute a common risk factor in the development of gentle and severe FP, ATD might not be sufficient to cause severe FP episodes. Based on human (11) and animal studies (33), stress could be the main factor eliciting repetitive behavior. Our birds were not assigned to a specific stress treatment in the current experiment. Therefore, whether ATD alone was an insufficient stressor, and whether a more severe stressor in combination with ATD would increase the risk of severe FP in a group housing situation needs further investigation.

No differences in propensity to develop gentle or severe FP due to ATD were associated with genetic line. We hypothesized that the H genotype, selected for FP activity, would be more vulnerable, due to potential heritable neurochemical deficits (7), leading to higher numbers of FP bouts after ATD. While studies in humans point toward differential effects of ATD on behavior between healthy and unhealthy populations (34), we did not observe that birds of the H genotype constituted a vulnerable population that responded more sensitively to ATD in their performance of FP in a social setting. However, a more severe ATD treatment and/or an additional stressor might have had induced higher rates of gentle and/or severe FP in these genetically susceptible individuals. However, it remains possible that the H birds developed FP under the ATD treatment first, which was observed and then mimicked by the rest of the group out of frustration (35). The rest of the group could also have been stressed by the active peckers running around to find recipients, with this inherent stressor causing them to begin pecking one another, in turn (36). However, whether the H birds started FP under the ATD treatment and triggered a chain reaction causing a shift in the behavior of their conspecifics, was not analyzed in the present study.

Overall, in the operant chamber, rewarded key pecks were unaffected by ATD/BC treatment or phenotype (pecker, recipient, bystander). This indicates that all birds (who were not food-deprived) were similarly motivated to obtain a high-quality food reward, irrespective of treatment. However, the maximum number of theoretically obtainable rewards (*n* = 32) within the 4-min testing period was not reached by any of the subjects (mean = 19.8 ± 2.1 SD, maximum = 26, minimum = 10) and this may give rise to the concern that some of the birds were not completely motivated to obtain rewards. Interestingly, video observations showed that rather than spending 2 s eating from the feeder, some hens learned to spend that time using their beaks to push the food out from the feeder onto the ground, in order to freely consume the food even after access to the feeder was removed. This might explain why the hens did not achieve the maximum number of rewards.

ATD increased the number of repetitive 5-s delay key pecks, with its most pronounced effect on P birds, and also increased the number of repetitive 5-s delay non-key pecks. Interestingly, environmental stress seems to be an important factor in the involvement of 5-HT in repetitive behavior (37). Our birds were subjected to a test situation in which they were only able to access a high-quality food reward for a short period of time by constantly moving and shifting their attention between the key and feeder. Considering this challenging procedure, the increased number of 5-s delay key and non-key pecks can be seen as stress-related repetitive pecking behavior. Additionally, from research conducted on humans, it is well-understood that ATD is more effective in vulnerable individuals, namely individuals with a past or present underlying dysfunction of their 5-HT system (38). Although, we did not measure 5-HT biochemical parameters in our birds, P birds may have been more vulnerable to the ATD treatment and/or the testing stress, explaining the higher number of 5-s delay key and non-key pecks performed by these birds. Additionally, it may be assumed that after ATD, P birds performed more poorly with the 5-s delay period compared to the BC treatment because they perceived the delay as a form of distractor stimulus from the reward. This suggests that ATD may have shifted their attention to the distracting delay period, causing them to peck at the perceived distractor—manifesting in this case as unrewarded pecks on and around the key and feeder. However, whether the higher number of pecks seen in P birds was due to this attentional shift toward a perceived distractor, or due to frustration, cannot be determined by the present results and requires further investigation.

While the present study is a good starting point into the investigation of the relationship between the 5-HT system and FP, it is limited by the fact that we did not collect and analyze baseline and ATD- or BC-related plasma TRP and metabolite levels. However, the method of ATD treatment that we used has been previously shown to robustly decrease blood TRP in laying hens 3 h after application (23). With regards to the magnitude and duration of effects of the dietary ATD treatment, it needs to be noted that a more prolonged depletion might have led to more pronounced effects in the group housing situation and/or during the operant learning task. However, the present study adds important starting-point data that allow for future studies involving prolonged TRP depletion. Additionally, brief dietary ATD treatment procedures may not capture the potentially complex relationships between the 5-HT system and behavior. Likewise, we need to consider that “direct evidence that ATD decreases extracellular 5-HT concentrations is still lacking in mammals, and that several studies provide support for alternative underlying mechanisms of ATD in mammals,” as recently outlined by van Donkelaar et al. (39) and Young (25). Similarly, the same consideration should be made for avian species (23), calling for caution when attributing the ATD results solely to 5-HT mechanisms.

In summary, although the ATD technique has been used extensively in psychiatric research over recent decades, and it continues to be a popular method in behavioral disorder studies of the 5-HT system in humans and mammals, the present study detected, for the first time, an effect of dietary ATD on repetitive pecking behavior in laying hens living in a group setting, as well as their operant learning behavior. In the future, ATD could be of potential value to further disentangle the complex interplay between the 5-HT system and alternative neurobiological mechanisms, its dietary manipulation by ATD treatments, and FP in laying hens.

## Ethics Statement

This study was approved by the University of Guelph Animal Care Committee before testing (Animal Utilization Protocol # 3206).

## Author Contributions

AH-M, PB, JK, WK, and PF conceived and designed the study. PB, JC, and PM carried out the study. JC and PM analyzed the videos. PB analyzd the data with help of Dr. Michelle Edwards (UoG statistician). PB and JC drafted the manuscript. AH-M, PM, JK, WK, and PF contributed to writing the manuscript. All authors read and approved the final manuscript.

### Conflict of Interest Statement

The authors declare that the research was conducted in the absence of any commercial or financial relationships that could be construed as a potential conflict of interest.

## Abbreviations

BC: Balanced Control
FP: Feather Pecking
H: High Feather Pecking Behavior
L: Low Feather Pecking Behavior
P: Feather Pecker
UC: Unselected Control Line.
